# Microbiological Profile in Patients Having Keratitis in a Tertiary Care Hospital in India

**DOI:** 10.7759/cureus.31653

**Published:** 2022-11-18

**Authors:** Pritha Pramanick, Mallika Sengupta, Madhumita Banerjee, Sougata Ghosh, Anita Nandi Mitra, Mandira Chakraborty, Manideepa Sengupta

**Affiliations:** 1 Infectious Disease, Medical College and Hospital, Kolkata, IND; 2 Infectious Disease, All India Institute of Medical Sciences, Kalyani, IND; 3 Ophthalmology, Medical College and Hospital, Kolkata, IND

**Keywords:** microbiological culture, fungus, staphylococcus, keratitis, corneal ulcer

## Abstract

Background

Corneal ulcer or keratitis is defined as a loss of corneal epithelium with underlying stromal infiltration and suppuration associated with signs of inflammation. Corneal blindness is a significant public health problem worldwide; infectious keratitis is one of the predominant preventable causes of blindness. Several studies have evaluated microbial infectious keratitis's etiology, management, and outcome. However, there are regional variations in corneal ulcers' prevalence, risk factors, and outcome. The objective of this study was to isolate and identify the bacterial, fungal, viral, and protozoal etiological organisms causing infectious corneal ulcers along with their prevalence and antimicrobial sensitivity pattern.

Methods

A prospective observational study was done in the Department of Microbiology and RIO, Medical College & Hospital, Kolkata, for a period of 1 year (February 2019 to January 2020) after obtaining clearance from the Institutional Ethics Committee. Informed consent, demographic data, history of disease onset, duration of symptoms, associated co-morbidities, etc., were taken from the patients fulfilling the inclusion criteria. Corneal scraping samples were collected sterilely to detect bacterial, fungal, parasitic, and viral isolates and identified by standard laboratory procedures.

Results

A total of 80 patients were included in the study. The risk factors included foreign body in 24 (30%), blunt trauma in 10 (12.5%), steroid use in 8 (10%), contact lens user 4 (5%), and spontaneous in 34 (42.5%). Among these 80 patients, 18 showed growth of bacteria, including *Staphylococcus aureus, Streptococcus pneumoniae, Streptococcus pyogenes*, and *Pseudomonas aeruginosa*; four had growth of fungi, including *Aspergillus spp*. and *Fusarium spp, *and two were positive for Herpes simplex virus by IFA.

Conclusion

Early diagnosis and prompt keratitis treatment are critical for preventing visual loss. The identification of the various causative agents of keratitis is essential for the proper management of the cases.

## Introduction

A corneal ulcer is a loss of the cornea's epithelium, which may be accompanied by underlying stromal infiltration, pus formation, and inflammation [1]. Losing vision due to the cornea being affected is an important health problem. A corneal ulcer caused by infectious agents is a common cause of blindness which can be prevented by appropriate management at the proper time [2]. The organisms enter mainly through a breach in the corneal epithelium. There may be a disruption in the surface epithelium, decreased resistance of the corneal epithelium, or any factors like injury, diabetes, etc., leading to necrosis or desquamation. There are different predisposing factors like the introduction of organisms during trauma, prolonged use of topical steroids, dry eyes, wearing of contact lenses, and poor ocular hygiene, which may lead to an increased risk of corneal ulcer [3].

Corneal opacity is a significant cause of blindness worldwide, affecting approximately 6 million people globally. It is also responsible for 1.5-2 million new cases of blindness per year. A corneal ulcer's etiology includes infection, inflammation, trauma, degeneration, and nutritional deficiency. Infectious keratitis is one of the world's most common causes of corneal blindness, with an estimated incidence ranging from 2.5 to 799 per 100,000 population-year. Bacteria, fungi, viruses, and parasites can cause infectious keratitis. Depending upon the geographical location, bacteria and fungi be the most common causative microbial agents for corneal infection. Worldwide, parasitic and viral keratitis are less common, but still, they are important causes of corneal blindness in developed countries. Contact lens usage, eye injury, ocular surface diseases, lid diseases, and post-ocular surgery are known major risk factors for infectious keratitis. Topical antimicrobial agents having a wide range of action is the current mainstay of treatment for infectious keratitis. However, the emergence of antimicrobial resistance, especially multidrug resistance, is leading to difficulty in managing these cases [4].

The causative agent of a corneal ulcer is variable in different geographical regions. The bacterial etiological agents associated with corneal ulcers include Staphylococcus aureus, Staphylococcus epidermidis, Streptococcus pneumoniae, Streptococcus pyogenes, Moraxella species, Pseudomonas aeruginosa, Proteus species, Klebsiella pneumoniae, and Escherichia coli. The common fungi associated with keratitis include Candida albicans, Aspergillus flavus, Fusarium spp., Penicillium species, and Aspergillus fumigatus, while the common parasites include Acanthamoeba spp. Herpes Simplex Virus type 1 (HSV-1) is the most common agent of viral corneal ulcers. In addition, Pseudomonas spp. is an opportunistic bacteria linked with keratitis from contact lenses [5].

The most typical clinical sign in bacterial keratitis seen most frequently is unilateral and, in some cases, presents with bilateral ocular pain along with photophobia or abnormal sensitivity to light. The disruption of corneal epithelium exposes corneal nerve endings. This contributes to the pain and discomfort seen in corneal ulceration. Typically, there is inflammation and congestion of the anterior segment of the eye. There is a thick, profuse mucoid or purulent discharge from the eyes. The eyelids are edematous and swollen, along with inflammation of the underlying palpebral conjunctivae. There is a focal area of stromal infiltrate with an overlying area of epithelial excavation in a corneal ulcer. The infiltrate is well-circumscribed with distinct borders. There is edema in the cornea and reduced visual acuity. Severe cases of bacterial keratitis lead to profound anterior chamber reaction and hypopyon, an accumulation of pus cells in the anterior chamber. Ciliary body inflammation sometimes causes hypotony or lower intraocular pressure. Inflammatory cells in the aqueous may also clog the trabecular meshwork and increase the intraocular pressure [6]. According to the Royal College of Ophthalmologists, the empirical therapy for bacterial corneal ulcers is with a broad-spectrum topical antibiotic. The most commonly used ones are the fluoroquinolones ciprofloxacin, moxifloxacin, levofloxacin, and ofloxacin [7].

The fungal agents of keratitis may be both yeasts and filamentous fungi. The typical presentation of the patients includes a red, painful eye and decreased vision. On examination, there is a marked conjunctival hyperemia seen as red eye, along with corneal infiltrates or corneal opacity, which looks whitish. Fungal keratitis is more likely if the ulcer has serrated margins and raised slough [8]. The first treatment for fungal keratitis is topical natamycin, although topical amphotericin B is the best choice for Aspergillus and Candida keratitis [9].

Viral keratitis presents as non-suppurative superficial keratitis. Among the various viruses causing keratitis, Herpes simplex virus and Adenovirus are the most prevalent, and these viruses are known to produce punctate epithelial erosions. More rarely, viruses like vaccinia, measles, mumps, and herpes zoster may affect the cornea. In developed countries, Herpes simplex virus keratitis is the most frequent cause of corneal ulceration and subsequent visual disability and blindness [10].

This study aimed to identify the common etiological agents of infectious corneal ulcer and their prevalence and determine antimicrobial susceptibility. 

## Materials and methods

Patients selection and sample collection

A prospective observational study was done in the Department of Microbiology and Regional Institute of Ophthalmology (RIO), Medical College & Hospital, Kolkata, for a period of 1 year (February 2019 to January 2020) after obtaining clearance from the Institutional Ethics Committee. The Institutional Ethics Committee approved the study via Reference no MC/ KOL/ IEC/ NON-SPON/ 175/12-2018 dated 22.12.2018. All patients with keratitis, including traumatic keratitis, exposure keratitis, neuroparalytic keratitis, and keratitis in contact lens users, were included in the study. Ocular diseases other than keratitis were excluded from the study. Informed consent from the patients or the parents of the patients in cases of children, demographic data, history of onset of disease, duration of symptoms, and associated co-morbidities were taken from the patients fulfilling the inclusion criteria. Corneal scraping samples were collected sterilely to detect bacterial, fungal, parasitic, and viral isolates and identified by standard laboratory procedures. Two drops of local anesthetic were given to the affected eye for taking corneal scrapings. Five minutes after the instillation of the local anesthetic agent, corneal scrapings were taken by sterile Bard Parker No. 15 scalpel blade under a slit lamp [11]. Care was taken to avoid touching the lashes or lids while obtaining the material from the base and the peripheral margins of the ulcer.

Tests performed

The corneal scraping was used for Gram staining, KOH preparation, wet preparation, and bacterial and fungal culture. The corneal scraping was streaked in a "C" shaped manner on blood agar, MacConkey's agar, and chocolate agar media and incubated aerobically at 370 C for a maximum of up to 48 hours; blood agar and chocolate agar were incubated in 5-10% CO2 incubator and Mac Conkey's agar in ambient air. Organism growth was identified by standard microbiological techniques, including Gram staining, relevant biochemical reactions, and the VITEK 2 Compact instrument (bioMerieux Inc., France). Antibiotic susceptibility testing was performed on Mueller-Hinton agar plates using the Kirby-Bauer disc diffusion method and was interpreted according to the Clinical and Laboratory Standards Institute guidelines (2018 version) [12]. The Staphylococcus isolates were tested with cefoxitin (30 µg) as a surrogate marker for methicillin-resistant Staphylococcus aureus (MRSA), fluoroquinolones like ciprofloxacin (5 µg), levofloxacin (5 µg), aminoglycosides like amikacin (30 µg), gentamicin (10 µg), erythromycin (15 µg), clindamycin (2 µg), trimethoprim-sulfamethoxazole (1.25/23.75 µg), doxycycline (30 µg), and linezolid (30 µg) using the disc diffusion method and with vancomycin and teicoplanin using the E-strip method to determine the minimum inhibitory concentration for these two antibiotics. A D-test was performed with erythromycin and clindamycin discs according to the CLSI guidelines to determine inducible clindamycin resistance. For Pseudomonas spp., the isolates were tested against cephalosporins like ceftazidime (30 µg), cefepime (30 µg), beta-lactam with beta-lactamase inhibitor combination like piperacillin/tazobactam (100/10 µg), cefoperazone/sulbactam (75/30 µg), fluoroquinolones like ciprofloxacin (5 µg), levofloxacin (5 µg), aminoglycosides like amikacin (30 µg), gentamicin (10 µg), tobramycin (10 µg), carbapenems like meropenem (10 µg), imipenem (10 µg), and aztreonam (30 µg). For Streptococcus species, including Streptococcus pneumoniae and Streptococcus pyogenes, the isolates were tested against penicillin (10 µg), ciprofloxacin (5 µg), levofloxacin (5µg), erythromycin (15µg), clindamycin (2 µg), vancomycin (30 µg) and linezolid (30µg) and trimethoprim-sulfamethoxazole (1.25/23.75 µg) for Streptococcus pneumoniae only.

The corneal scraping was inoculated in Saboraud's dextrose agar (SDA) besides KOH preparation to look for fungus growth and incubated at 250C and 370C and observed daily for the first seven days and on alternate days for the next 14 days to look for growth of fungi. If no growth occurred after 21 days of incubation, the fungal culture was declared negative. To identify fungal species, the colony on SDA was used for lactophenol cotton blue tease mount, and the fungus morphology was studied. For the identification of parasites, a direct lactophenol cotton blue preparation was done from the sample to look for the Acanthamoeba trophozoite and cyst.

For identification of herpes keratitis, the samples negative for bacterial culture and fungal smear were only included. Scraped material was smeared on two glass slides for Giemsa stain and immunofluorescent assay (IFA). The smear for Giemsa stain was stained by Giemsa stain for the observation of multinucleate giant cells with or without lymphocytes and intranuclear inclusions. Smears for immunofluorescence assay were fixed in cold acetone, and IFA was done according to the kit instructions of Agilent (Dako, Denmark). The slides were assessed under a reflected light fluorescence microscope at 490nm. Positive staining for HSV was represented by the presence of one or more basal epithelial cells exhibiting specific bright apple green fluorescence. The absence of fluorescence indicated a presumptive negative result [13].

All data were entered in the Excel spreadsheet. Data were summarized using mean with standard deviation for continuous variables and frequency with percentages for categorical variables.

## Results

**Figure 1:**
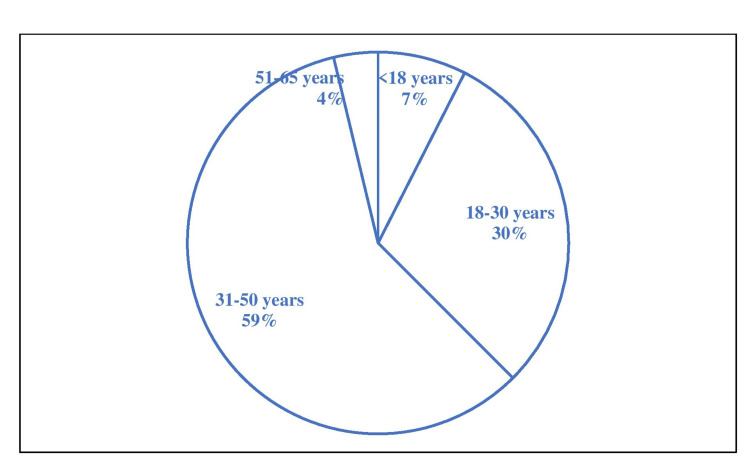
Age distribution of patients included.

A total of 80 patients were included in the study for one year (February 2019 to January 2020). The most affected age group was 31 - 50 years, with 47 (58.75%) patients, followed by 24 (30%) patients in 18 - 30 years, as shown in Figure 1.

The lowest age was 11, and the highest age was 64 years. Among the patients included in the study, 58 (72.5%) were males, and 22 (27.5%) were female patients. The most common occupation among the patients included was farmer 34 (42.5%), as shown in Table 1.

**Table 1:** Occupation of the patients included (n=80)

Occupation	No (%)
Farmer	34 (42.5%)
Driver	16 (20%)
Student	14 (17.5%)
Shop keeper	7 (8.75%)
Housewife	6 (7.5%)
Unemployed	3 (3.75%)

The predisposing factors included foreign body in 24 (30%), blunt trauma in 10 (12.5%), steroid use in 8 (10%), contact lens user 4 (5%), and spontaneous or unknown in 34 (42.5%) (Table 2). Among the different foreign bodies, exposure included animal tail injury, pollen, insect, iron particle, and mud exposure, among others. Diabetes and hypertension were the co-morbidities noted in these patients.

**Table 2:** Risk factors for the patients included (n=80)

Risk factor	No. (%)
Foreign body	24 (30%)
Blunt trauma	10 (12.5%)
Steroid use	8 (10%)
Contact lens user	4 (5%)
Spontaneous or unknown	34 (42.5%)

**Figure 2:**
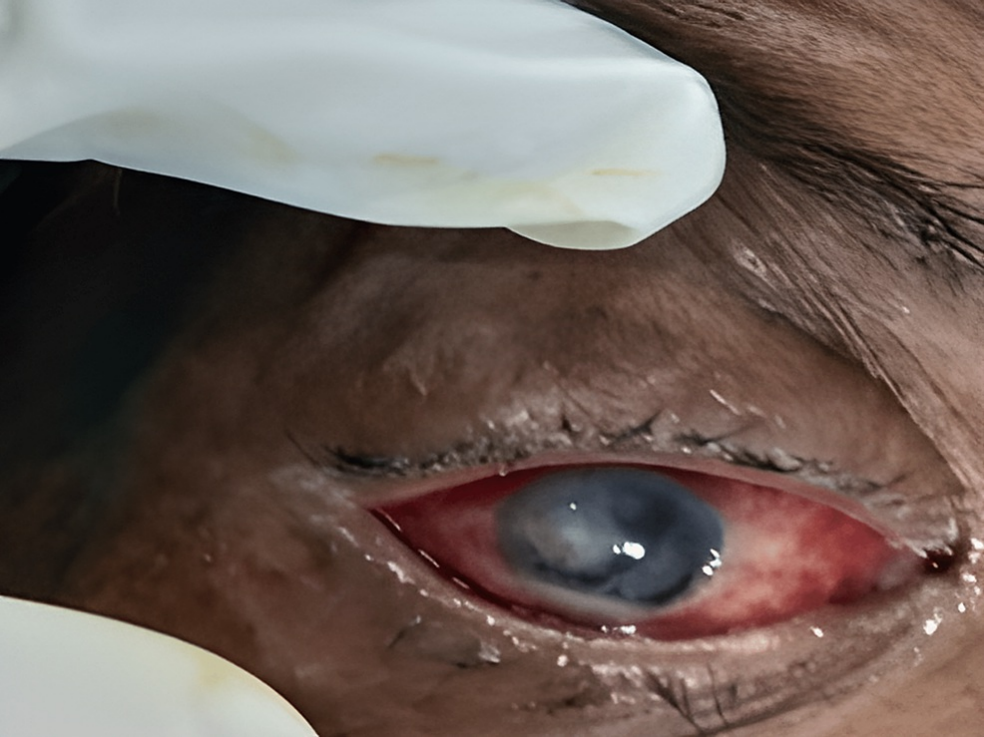
Fungal corneal ulcer

**Figure 3:**
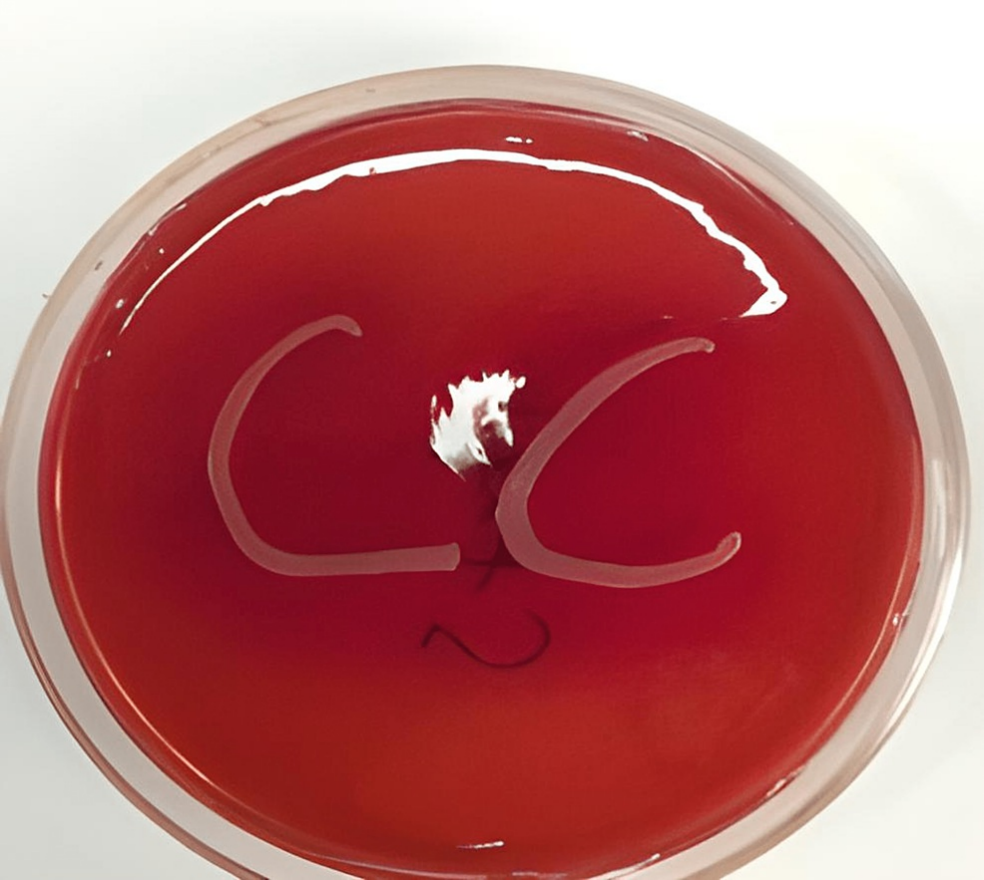
Growth of Staphylococcus aureus from corneal scraping

**Figure 4:**
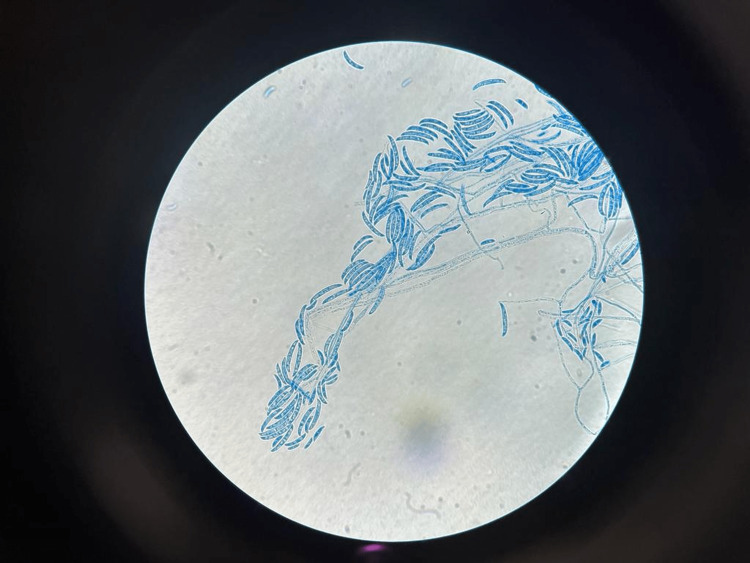
Microscopic picture of Fusarium spp. grown in culture

Among these 80 patients, 18 showed growth of bacteria, including Staphylococcus aureus, Streptococcus pneumoniae, Streptococcus pyogenes, and Pseudomonas aeruginosa; four had growth of fungi, including Aspergillus spp. and Fusarium spp. and two were positive for Herpes simplex virus by IFA. Two samples showed Gram-positive cocci in Gram stain but did not yield any bacterial growth in culture. In total, 26 (32.5%) patients had microbiological confirmation of the causative agent, and other samples did not yield any pathogen. The distribution of growth of different organisms is given in Table 3. Figure 2 shows a fungal corneal ulcer. Figure 3 shows the growth of bacteria in an inoculated culture plate. Figure 4 shows a microscopic picture of a Fusarium grown in a culture.

**Table 3:** Organisms demonstrated from the keratitis patients

Organism	Method of demonstration	No
Staphylococcus aureus	Bacterial culture	12
Streptococcus pneumoniae	Bacterial culture	3
Streptococcus pyogenes	Bacterial culture	2
Pseudomonas aeruginosa	Bacterial culture	1
Gram-positive cocci	Only Gram stain	2
Aspergillus spp.	Fungal culture	3
Fusarium spp	Fungal culture	1
Herpes simplex virus	IFA	2

Among the 18 bacterial growths isolated in culture, 17 were Gram-positive cocci. All staphylococci were susceptible to vancomycin, teicoplanin, and linezolid and most susceptible to aminoglycosides, doxycycline, and fluoroquinolones. There were 4 (33.3%) cases of methicillin-resistant S. aureus (MRSA) (Table 4).

**Table 4:** Antibiotic susceptibility of the isolated Gram-positive cocci

Antibiotic	S. aureus (n = 12)	Streptococcus pneumoniae (n = 3)	Streptococcus pyogenes (n = 2)
Penicillin	NA	3 (100%)	2 (100%)
Cefoxitin	8 (66.7%)	NA	NA
Erythromycin	7 (58.33%)	2 (66.67%)	1 (50%)
Clindamycin	9 (75%)	3 (100%)	2 (100%)
Ciprofloxacin	9 (75%)	3 (100%)	2 (100%)
Levofloxacin	10 (83.3%)	3 (100%)	2 (100%)
Gentamicin	11 (91.7%)	NA	NA
Amikacin	11 (91.7%)	NA	NA
Doxycycline	11 (91.7%)	NA	NA
Cotrimoxazole	10 (83.3%)	3 (100%)	NA
Linezolid	12 (100%)	18 (100%)	2 (100%)
Vancomycin	12 (100%)	18 (100%)	2 (100%)
Teicoplanin	12 (100%)	NA	NA

Only one P. aeruginosa isolate was susceptible to cefepime, piperacillin/tazobactam, cefoperazone/sulbactam, imipenem, meropenem, ciprofloxacin, levofloxacin and resistant to ceftazidime, amikacin, tobramycin, and gentamicin.

## Discussion

The study was done to isolate and identify the bacterial, fungal, viral, and protozoal etiological agents of infectious corneal ulcers and their prevalence. A total of 80 patients were included in the study for one year (February 2019 to January 2020).

In this study, the most affected age group was 31 - 50 years, with 47 (58.75%) patients, followed by 24 (30%) patients in 18 - 30 years. This finding is like that of a study conducted by Yewale et al., where the predominant age group was 21-40 years with 44 (55%) followed by the age group of 41-60 years 19 (23.75%) [14]. Another study by Gupta and colleagues observed a slight variation in age distribution where the predominant age group was 41-50 years (29.16%) followed by 31- 40 years (22.9%) [15].

Among the patients in the present study, 58 (72.5%) were males, and 22 (27.5%) were female. In a review by Gopinathan et al., among patients with keratitis, there was similar gender distribution, with 69.3% of patients being male and 30.7% of patients being female [16]. However, a study by Ibrahim YW et al. from the UK showed a different trend of gender distribution as female predominance was found. In their study, there were 54.5% female patients, which outnumbered the male patients (45.5%). Female preponderance in this study was primarily due to the use of contact lenses among females [17]. However, another study done in Pakistan showed that (61.5%) of patients were female and 38.4% of patients were male among the 65 cases of infective keratitis included in the study [18]. Hence, the gender distribution among the cases of corneal ulceration may vary depending on the country in which the study is being conducted.

In the current study, the most important predisposing factors included foreign body in 24 (30%), blunt trauma in 10 (12.5%), steroid use in 8 (10%), contact lens user 4 (5%) and spontaneous or unknown in 34 (42.5%). The foreign body exposure included animal tail injury, pollen, insect, iron particle, and mud exposure. Similar results were obtained by Kursiah et al., where foreign body injury was encountered in 32% and trauma in 25% of the included population, implicating foreign body injury is one of the most important causes of the corneal ulcer [19]. A study in China found a prevalence of 0.13% of infective keratitis among bandage contact lens wearers [20]. In another study done in Thailand to look for the different types of ocular injury, it was found that traumatic corneal ulcer was more common in closed globe injury. Corneal penetration and foreign body in the eye were common causes of open globe injury [21]. In a study done by Puig and group in South Texas, USA, the most common risk factors for infectious keratitis were the usage of contact lenses (32.4%), underlying corneal disease (17.6%), trauma (14.3%), and ocular surface disease (13.7%) [22].

Among these 80 patients in our study, 18 showed growth of bacteria, including Staphylococcus aureus, Streptococcus pneumoniae, Streptococcus pyogenes, and Pseudomonas aeruginosa, four had growth of fungi, including Aspergillus spp. and Fusarium spp. and two were positive for Herpes simplex virus by IFA. Two samples showed Gram-positive cocci in Gram stain but did not yield any bacterial growth in culture. This was most probably due to the use of topical antibiotics, which are available over the counter. In total, 26 (32.5%) patients had microbiological confirmation of the causative agent, and other samples did not yield any pathogen.

The study by Rajini et al. reported that among 117 culture-positive cases of corneal ulcers, bacterial isolates contributed to 52 (44.5%) and fungal isolates contributed to 58 (49.5%). The most prevalent bacterial isolates included Staphylococcus aureus among the Gram-positive bacteria and Pseudomonas aeruginosa among the Gram-negative bacteria. Fungal isolates included Fusarium 31% followed by Aspergillus sp. 11% [23].

In a study done in Kathmandu, Nepal, bacterial isolates were obtained in 63.2%, and fungal isolates were obtained in 6.7% with corneal ulcers and growth of both fungi and bacteria revived from 10.1% of cases. Among the 398 bacterial isolates included in the study, 31.1% were Streptococcus pneumoniae, the most typical isolated organism in their study, followed by Staphylococcus epidermidis, Staphylococcus aureus, and Pseudomonas species. Among the 68 positive fungal isolates, 47.0% were Aspergillus species, followed by Candida and Fusarium species [24].

In an epidemiological study done in Gangetic West Bengal, from 2001 to 2003, 1198 patients with suppurative keratitis were evaluated. Their study found that the most common predisposing factor for keratitis was ocular trauma, seen in 82.9% of patients, followed by topical corticosteroids in 19.28% of patients. There was growth in bacterial and fungal cultures in 67.7% of patients. Among these culture-positive cases, 62.7% of patients had pure growth of fungi, 22.7% had pure growth of bacteria, and 14.1% had mixed fungal with bacterial infections. Acanthamoeba was detected in 0.49% of patients. The most common fungal pathogen was Aspergillus spp, followed by Fusarium spp., which is like the findings of our study. The most common bacterial isolates were Staphylococcus aureus, followed by Pseudomonas spp. [2].

In a study done at a public hospital in Australia over five years, from October 1999 to September 2004, the different risk factors, demographic data, and etiological agents of keratitis were studied. A total of 253 corneal scrapings from 231 patients were included in the study. Gram-positive bacteria, including Staphylococcus and Streptococcus, were the most frequent group of organisms isolated (29%). Of the Gram-negative isolates (23%), Pseudomonas aeruginosa was the most frequent isolate. In 5% of cases, fungi were isolated. Among the fungi, Fusarium was the most widely prevalent. There was also a significant variation in the monthly recovery of the different organisms. Pseudomonas aeruginosa and fungi were more frequently isolated in the summer months, whereas Streptococcus pneumoniae was more frequently isolated in the winter season [25].

A study was carried out in Rajshahi Medical College Hospital, Dhaka, Bangladesh, to isolate and identify the causative etiological agents of infective keratitis and to look for the antimicrobial susceptibility pattern of these bacterial isolates. Fifty-six patients were included in their study, and corneal swabs and scraping were collected aseptically from suppurative corneal ulcers. There was growth seen in a total of 47 (83.93%) cases, with pure fungal growth in 24 (42.86%), pure bacterial growth in 14 (25.0%), and mixed bacterial and fungal growth seen in 9 (16.07%) cases. Among the fungal isolates, Aspergillus fumigatus was the most frequently isolated agent, followed by Fusarium spp, Mucor spp, Aspergillus flavus, Aspergillus niger, Rhizopus spp, and Alternaria spp. Staphylococcus aureus was the predominant bacterial pathogen, followed by Pseudomonas spp., H. influenzae, Staphylococcus epidermidis, Streptococcus pneumoniae, and E. coli. Lomefloxacin, tobramycin, and gentamicin were mainly susceptible to most bacterial pathogens isolated, as demonstrated by in-vitro susceptibility testing [26].

A retrospective review of all cases presenting with keratitis at the cornea clinic, Aravind Eye Hospital, Coimbatore, from August 1997 to July 2003, a period of 6 years, was done for screening patients with a provisional diagnosis of Acanthamoeba keratitis. Only the cases with culture-positive Acanthamoeba keratitis were included in the analysis of the study. From a total of 4519 patients who came to the hospital, 32 patients were confirmed to be positive for Acanthamoeba keratitis by isolation in culture. The majority of these Acanthamoeba keratitis cases (54.2%) had a history of corneal trauma by solid objects, which was a significant risk factor for the development of corneal ulceration [27].

A study in North India showed that the various risk factors for corneal ulcers were trauma and the injudicious use of topical antibiotics and corticosteroids. In a 6-year study done by Chander et al. in North India to look for the fungal etiology of corneal ulcers, among the 730 patients included in the study, fungi were detected in 61 (8.4%) cases only. Aspergillus spp. was the most frequently isolated fungus accounting for 40.1% of the total fungal isolates, followed by Fusarium spp., Curvularia spp., Candida albicans, Acremonium spp., Paecilomyces spp., Penicillium spp., Alternaria spp., Fonsecaea pedrosoi var. Cladosporium, Pseudallescheria boydii, Drechslera spp., and Aureobasidium pullulans [28]. In a recent study done on fungal keratitis in North India and North-East India, Aspergillus sp. (52.1%) and Fusarium sp. (47.61%) were the predominant etiological agents isolated from cases in North and Northeast India, respectively [29].

In a study done in Maharashtra, it was found that fungal corneal ulcer was more prevalent than other forms of infectious keratitis. From January 2015 to February 2017 total of 680 patients attended the cornea clinic of the hospital. Among these patients, 88 were diagnosed of having infective keratitis. Most of the included group were 41 - 60 years of age, followed by 21-40 years. There was a higher prevalence of keratitis among males (61%) compared to females. Fungal keratitis (59.09%) was more prevalent than bacterial (19.31%) and viral (17.04%) keratitis. Ocular trauma and injury were the most common (42%) among farmers [30]. These findings are very similar to the findings of our study. However, the current study had more bacterial pathogens than fungi.

Among the 18 bacterial growths isolated in culture in the present study, 17 were Gram-positive cocci. All staphylococci were susceptible to vancomycin, teicoplanin, and linezolid and most susceptible to aminoglycosides, doxycycline, and fluoroquinolones. There were 4 (33.3%) cases of methicillin-resistant S. aureus (MRSA). In a review by Chang et al., it was found that 30.7% of all Staphylococcus aureus isolates were MRSA. All these isolates were susceptible to vancomycin [31]. Topical linezolid is a very effective treatment option for keratitis caused by Gram-positive organisms, as shown by Tu and Jain [32]. Another study by Galvis et al. also reinforced the utility of using linezolid topical antibiotics for Staphylococcus isolates [33].

The limitations of the current study included that the antifungal susceptibility testing for the isolated fungi was beyond the scope of the study. Moreover, most patients included in the study had a resolution of symptoms and did not come back for follow-up. Among the viral etiologies for corneal ulcers, only screening was done for the herpes simplex virus. 

## Conclusions

Early diagnosis and prompt treatment of keratitis are critical for preventing visual loss. The identification of the various causative agents of keratitis is essential for the proper management of the cases. As there are various etiologies of infectious keratitis, it is essential to know the causative agent for treating these patients.
